# The Nudge Effect of “Happy to Chat” Badges: Evidence from England

**DOI:** 10.1007/s10902-026-01057-9

**Published:** 2026-06-05

**Authors:** Dorothy A. Yen, Jung min Jang, Jingshan Yang, Ming-yao Jen, Christina Victor

**Affiliations:** 1 Brunel Business School, Brunel University London, Uxbridge, UB8 3PH UK; 2 Birmingham City Business School, Birmingham City University, Birmingham, B4 7BD UK; 3 Brunel University London, Uxbridge, UB8 3PH UK

**Keywords:** Happy to chat, Badge, Nudge, Social connection, Talking to strangers, Social isolation

## Abstract

Social connection is essential for well-being, yet people often avoid interacting with strangers due to concerns about conversation quality or a lack of shared interests. This study investigates whether the *Happy to Chat* badge can act as a behavioural nudge to promote social connectedness. Using a nationally representative online sample of 1,738 adults in England (via YouGov), we tested whether wearing the badge influences perceptions of friendliness, trustworthiness, and interest in conversation, as well as social behaviours such as smiling, eye contact, nodding, and willingness to initiate a chat. Results show that badge wearers are perceived as significantly more friendly, trustworthy, and open to conversation, and are more likely to receive social acknowledgements, although wearing the badge did not significantly increase others’ intention to initiate conversation. Age and gender effects were also observed, with older and female targets generally receiving more favourable social responses. These findings suggest that while the badge effectively promotes social recognition, it may be insufficient on its own to overcome deeper psychological barriers to initiating conversations with strangers. This paper confirms the effectiveness of the *Happy to Chat* badge as a nudge to promote social connectedness within communities and highlights its practical implications.

## Introduction

Social connection is widely recognised as a fundamental determinant of human wellbeing. Research consistently shows that individuals who spend more time interacting with others and less time alone report higher levels of subjective wellbeing (Diener & Seligman, 2002; Mehl et al., 2010). Staying connected and being recognised by others as members of society is conducive to one’s feeling of happiness (Diener & Seligman, 2002). Importantly, this feeling of connection does not only come from interactions and relationships with strong ties such as family and close friends, but also from interactions with weak ties, i.e. acquaintances and strangers (Gunaydin et al., 2020; Sandstrom & Dunn, 2014; Yen et al., 2024). While people tend to underestimate the enjoyment of talking to strangers (Schroeder et al., 2022), research has shown that brief encounters with strangers, e.g. saying hello, greeting each other in the park, engaging in brief everyday conversations, etc. can enhance subjective wellbeing and foster a sense of social connection and belongingness (Algoe, 2012; Keohane, 2021).

Nevertheless, it is not always easy for people to embrace the possible interactions with strangers. Studies (e.g. Sandstrom et al., 2021; Schroeder et al., 2022) show that people often shy away from talking to strangers, fearing that they would not enjoy the conversation, not like their conversation partner or worry about their own lack of conversational skills. They may also worry about their conversation partners not liking them, not finding the conversation enjoyable or not being interested in having a conversation (Keohane, 2021). As a result, individuals may miss opportunities to strengthen social connection and enhance subjective wellbeing., This issue is particularly salient given that approximately one-third (33%) of adults report experiencing loneliness worldwide (Ipsos, 2021), a situation potentially intensified following COVID-19 social distancing practices (ONS, 2022).

Against this background, this study examines whether a simple social signal—the “Happy to Chat” badge—can function as a social nudge that reshapes how strangers are perceived in everyday public encounters. Specifically, we ask whether signalling openness to interaction through a “Happy to Chat” badge influences social perception and behavioural intentions toward the wearer. Importantly, the “Happy to Chat” badge is not limited to individuals experiencing loneliness. While loneliness is often framed as an individual condition or problem, everyday social interaction is also shaped by broader social norms governing behaviour in public spaces and by shared expectations about when interaction with strangers is socially permissible (Simões Aelbrecht, 2016). The Happy to Chat initiative therefore adopts a universal rather than targeted approach. Instead of identifying vulnerable individuals, the badge functions as a shared social signal that normalises openness to interaction and legitimises everyday social engagement among strangers.

Using a nationally representative online sample of 1,738 participants in England—recruited through the survey company YouGov—we test the effectiveness of the Happy to Chat badge through a between-subjects experimental design. In particular, we investigate whether the badge leads wearers to be perceived as friendlier, more trustworthy, and more interested in having a conversation. We also explore whether badge wearers are more likely to receive desired forms of social acknowledgement—such as smiles, eye contact, or head nods—and to be approached for conversation, compared to individuals not wearing a badge. By examining both perceptual evaluations and behavioural intentions, the study contributes to emerging research on social nudges by demonstrating how universal social signals can lower initial psychological barriers to interaction and facilitate everyday social acknowledgement among strangers.

## Literature Review

### Talking to Strangers and Well-Being

Social isolation refers to the absence of interactions or contacts between individuals and their social network, which can often trigger loneliness, an unpleasant feeling due to a lack of companionship (Peplau & Perlman, 1982; de Jong Gierveld, 1998; Cattan et al., 2005). Feeling of loneliness is not just a negative emotion; abundant research has proved the negative relation between loneliness and medical issues such as risk of dementia, cognitive decline and cardiovascular disease (Boss et al., 2015; Valtora et al., 2016; Wilson et al., 2007). Unfortunately, the COVID-19 pandemic seems to have worsened the situation (Atzendorf & Gruber, 2022; Yen, et al., 2021), due to policies on quarantine and social distancing, implemented in most countries to control the spread of the disease. These interventions, by enforcing physical distance between individuals, mimic conditions similar to social isolation. According to the Office of National Statistics (ONS, 2022), 32% of adults reported always or often maintaining social distancing even in 2022, when COVID-19 was no longer considered a major threat.

One solution to reduce social isolation is to focus on increasing opportunities for social connections. Studies have shown that spending more time talking to others and less time being alone is pro-social and such behaviour is associated with higher well-being levels (Diener & Seligman, 2002; Lee et al., 2024; Mehl et al., 2010). For example, Mehl et al., (2010) discovered that substantive conversations, as opposed to superficial small talk, could lead to higher well-being, though their findings lack causal clarity. Other studies have shown that minimal social interactions with strangers could also contribute to subjective well-being (Gunaydin et al., 2020). For example, Sandstrom and Dunn (2014) report in their study with Starbucks drinkers in Canada that those who engaged in a social interaction with their barista experienced more positive emotions than those who simply ordered their coffee as efficiently as possible. Although they did not expect it, interacting with their baristas through smiles, eye contacts and brief conversations actually made those participants feel happier and created a sense of belonging. A similar study with commuters in London also found the same, people undervalue the enjoyment of talking to strangers, despite the results that commuters assigned to talk to strangers enjoyed their commute more so than those who did not talk to strangers (Schroeder et al., 2022). According to Algoe (2012), these brief encounters with strangers enhance subjective well-being by allowing people to recognise and appreciate the minor benefits of their daily interactions, using the find-remind-and-bind theory. This, in turn, fosters a sense of social connection and belongingness.

Data from a series of field and laboratory experiments conducted by Epley and Schroeder (2014) suggest that people often avoid talking to strangers because they mistakenly assume that such interactions will be unpleasant and that solitude will be more enjoyable. However, their findings showed that commuters reported more positive experiences when engaging with strangers than when sitting alone. Similarly, Zeeb and Joffe (2021) interviewed residents in the UK’s two largest cities and found that people categorise strangers as either ‘good’ (e.g., friendly, vulnerable, potentially interesting or likeable) or ‘bad’ (e.g., dangerous and/or rude). While interactions with ‘good’ strangers are viewed as enjoyable and enriching, positively influencing well-being, interactions with ‘bad’ strangers can evoke discomfort and perceived threat, potentially leading to distress.

The context of encounters—shaped by factors such as technology, setting, and time—significantly influences these perceptions. For example, strangers met in semi-public settings like shops or stadiums are generally perceived positively, whereas those lingering in poorly lit or deserted public spaces at night and displaying anti-social behaviours (e.g., shouting, swearing, smoking, or appearing intoxicated) may be perceived as threatening (Zeeb & Joffe, 2021). However, not all places at night are deemed as dangerous. Rosenfeld and Thomas (2012) pointed out that certain night-time environments, such as bars, clubs, and festivals, have actively facilitated positive interactions with strangers, particularly for socialising and dating purposes, underscoring the complexity of context-specific perceptions. Additionally, Sandstrom et al. (2022) conducted a week-long “talking to strangers” intervention, demonstrating that repeated positive experiences of engaging with strangers can improve individuals’ well-being by reducing fears of rejection, increasing conversational confidence, and decreasing awkwardness.

Atir et al. (2023) developed a framework identifying obstacles to connecting with strangers – intention, competence and opportunity, highlighting the need to promote conversations among strangers by showing one’s intention to connect. With abundance research and evidence showing that talking to strangers could lead to better well-being, why are people not doing it more often? People often avoid talking to strangers due to various fears, such as not being able to enjoy the conversation, not liking the person they are speaking with, or lacking conversational skills (Sandstrom et al., 2021). Studies have found that, in addition to worrying about not enjoying the conversation themselves, people also worry that the strangers they speak to may not find the conversation enjoyable. Schroeder et al. (2022) further showed that people often avoid initiating conversations with strangers because they mistakenly assume others are not interested in social interaction. Their experiments demonstrated that, although commuters frequently refrain from talking due to this misperception, those who did engage in conversation with strangers reported significantly more positive experiences than individuals who remained in solitude or in control conditions. This indicates that interventions could be developed to facilitate accurate perceptions of mutual interest in social interactions.

Multiple studies have also tested the relationship between extraverted behaviour and well-being and found that acting extraverted (bold, spontaneous, assertive and talkative) increases well-being while acting introverted (timid, withdrawn, inhibited and unadventurous) decreases well-being (Fleeson et al., 2002; Margolis & Lyubomirsky, 2019; McNiel & Fleeson, 2006; McNiel et al., 2010). These findings highlight the importance of figuring out and designing effective interventions that promote conversations with strangers to enhance not only the individuals’ well-being but also the community’ social well-being collectively.

Beyond individual motivations, everyday interaction with strangers is strongly governed by social norms regulating behaviour in public spaces. Goffman’s (1972) concept of civil inattention suggests that individuals often avoid acknowledging strangers not due to disinterest, but because non-engagement is interpreted as socially appropriate behaviour (Hirschauer, 2005). Consequently, the barrier to interaction may lie less in personal unwillingness and more in the absence of socially legitimate signals indicating openness to engagement.

### Nudge Intervention

Over the past decade, nudge theory has gained increasing popularity as an alternative method for promoting positive behavioural changes in citizens. Thaler and Sunstein provide a comprehensive definition of nudges:

“A nudge, is any aspect of the choice architecture that alters people’s behaviour in a predictable way without forbidding any options or significantly changing their economic incentives. Putting fruit at eye level counts as a nudge; banning junk food does not.” (Thaler & Sunstein, 2009, p.6).

Nudges operate by subtly restructuring choice environments without restricting freedom of choice, typically by increasing the salience or visibility of desirable options (Thaler & Sunstein, 2009). Such interventions guide behaviour through gentle prompts rather than coercion and have been applied across domains including finance, education, public safety, and social behaviour (Bovens, 2009; de Ridder et al., 2024). Extending this logic to social interaction, nudges may operate not only by changing individual choice architecture but also by making intentions visible to others. Signals that communicate openness, friendliness, or willingness to engage can reduce uncertainty in social encounters by clarifying mutual expectations of interaction.

Social interaction in public spaces is strongly shaped by implicit social norms. Goffman’s notion of civil inattention suggests that avoiding engagement with strangers is often interpreted as polite behaviour rather than social rejection (Hirschauer, 2005). In modern day society, people often have a prevention-focused mindset when navigating their everyday encounter with strangers. This leads individuals to remain cautious and avoid acknowledging others in public spaces (Keohane, 2021). Consequently, many individuals may be open to interaction but lack socially acceptable signals indicating mutual willingness to engage. Social nudges, such as the “Happy to Chat” badge, can therefore be understood as social signalling mechanisms that make prosocial intentions visible and shift perceived norms of interaction, thereby lowering coordination barriers between strangers rather than directly altering individual motivation. The social nudge also helps create a more enabling environment in which individuals experiencing loneliness may feel more comfortable approaching others and engaging in needed social interactions.

While Lucas et al. (2010) confirmed that subtle cues of social acceptance can prime a promotion-focused mindset in lonely people, decreasing their caution and encouraging social engagement, it highlights the potential to promote more social interactions in communities and societies through subtle cues. By using a subtle nudge, this type of intervention can remind people that talking to a stranger is a prosocial act (Sandstrom et al., 2021) and can also enhance their own wellbeing, contributing to feelings of happiness and reduced loneliness. For example, Yen et al. (2024) reveal that older people found these micro-conversations with strangers to be surprisingly rewarding and enjoyable. Rather than seeking personal gain, people often view these interactions as acts of kindness towards others, which in turn enhance their own sense of purpose and social connectedness (Yen et al., 2024).

Building on this theoretical framework, the present study examines whether wearing a “Happy to Chat” badge can function as an effective social nudge facilitating interaction among strangers. Rather than attempting to change individuals’ preferences, the intervention targets the coordination problem inherent in stranger interaction by signalling mutual willingness to engage (Atir et al., 2023). While Zeeb and Joffe (2021) show that people perceive ‘good’ strangers as friendly, interesting, and willing to connect—qualities associated with enjoyable and enriching conversations—the badge signals a person’s openness to connections, indicating their intention to be viewed as a ‘good’ stranger. Nevertheless, it preserves others’ freedom of choice, allowing them to decide whether or not they wish to acknowledge and interact accordingly (Thaler & Sunstein, 2021). The badge does not compel engagement but clearly signals the wearer’s openness to connect, legitimising a temporary suspension of civil inattention and potentially inviting a nod, smile, or conversation. In this way, it functions as a nudge that can positively shape how others perceive the wearer’s openness to social interaction while encouraging their own favourable behavioural responses. While the badge may also remind wearers of their willingness to engage, the present study focuses primarily on how the badge functions as a social signal interpreted by others,

Social perception research demonstrates that demographic cues such as age and gender systematically shape expectations of warmth, approachability, and trustworthiness (Fiske et al., 2018; Todorov et al., 2015). Older adults and women are often perceived as warmer social partners, which may independently influence willingness to engage (tel Stal et al., 2020). Including age and gender in the experimental design therefore allows examination of whether the proposed social nudge operates beyond established social stereotypes.

**Fig. 1:**
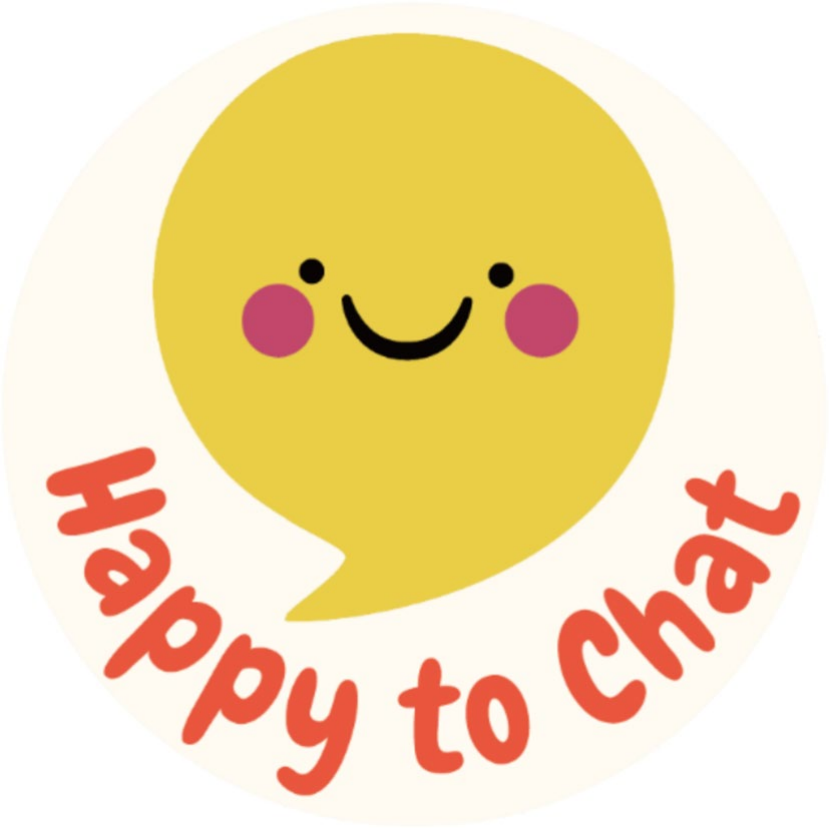
*Happy to Chat* badge

Building on this logic, the Happy to Chat badge addresses several well-documented barriers to social interaction: (1) It reduces social awkwardness. Research has found that a significant portion of the population feels awkward or shy when initiating conversations, such as making small talk. Wearing a “Happy to Chat” badge silently communicates that “I’m open to being approached for a chat.” This can reduce uncertainty about whether to initiate social interaction, as wearing the badge signals openness and legitimises being approached for conversation (Yen et al., 2024). (2) The badge conveys information saliently. It is visible and clearly signals the wearer’s willingness for interaction in an explicit manner. Earlier, we mentioned that people have misconceptions or false beliefs that strangers do not wish to talk, or that talking to strangers is intimidating and should be avoided (Sandstrom et al., 2021). The badge acts as a salient bridge to dismiss these misconceptions and false beliefs, serving as an invitation for conversations. (3) It challenges social norms and action tendencies. In large cities, civil inattention is often regarded as the norm, perceived as an act of thoughtfulness and politeness through self-distancing (Goffman, 1972; Keohane, 2021). Keohane (2021) presented real-life examples where items like an “I Talk to Strangers” T-shirt or a tote bag reading “Ask Me About the Book I’m Reading” enabled wearers to be acknowledged and engage in desired conversations. In this context, we want to explore whether wearing a *Happy to Chat* badge (see Fig. 1) can encourage people to reconsider the self-imposed social restriction of “not talking to strangers,” potentially establishing a new norm that benefits the society at large.

Thaler and Sunstein (2021) advocate marketers and other policymakers to nudge for good. Given the objective of making social connections easier by fostering a more welcoming and friendly community, the implementation of social nudges, such as the *Happy to Chat* badge, can be advantageous. Specifically, we question whether wearing the “Happy to Chat” message can nudge others to perceive the wearer as being friendlier (Hypothesis 1), more trustworthy (Hypothesis 2) and more interested in having conversations (Hypothesis 3). Moreover, we examine whether respondents are more likely to acknowledge and respond to individuals wearing *Happy to Chat* badges through social behaviours—such as making eye contact, smiling, or nodding (Hypothesis 4)—and whether they are more inclined to initiate conversations with the wearer (Hypothesis 5).

## Research Methods

The purpose of this study is to empirically evaluate the nudge effect of the Happy to Chat badge, specifically examining how the combination of the “Happy to Chat” text and smile image influences people’s perceptions of strangers and their willingness to acknowledge or engage in conversation with someone wearing the badge. To test this, we examined three conditions: (1) wearing the original badge featuring both the “Happy to Chat” message and a smile image, (2) wearing a badge displaying only the smile image (without the text), and (3) wearing no badge. Visual images of four strangers—varying in age and gender—were used as stimuli across these conditions. These stimuli reflected the intended social interactions promoted by the Happy to Chat campaign (see https://happytochat.uk for more details), with the aim of evaluating the effectiveness of the original badge design in eliciting a nudge effect.

### Stimuli Creation

For our experimental design, photo stimuli were created using Leonardo AI. We ordered the AI to create portraits of a friendly British older/mid-age, white man/woman, around 75–80/40–45 years old, full body, average beauty, casually dressed, British style, wearing an outdoor coat. Background: high details, in an old British Highstreet, cobblestone street, no traffic, with other shoppers in the background. These stimuli were generated to manipulate the variables of Age, and Gender across conditions. After the images were generated, we used photoshop to paste the badges. Badge presentation varied across three conditions: in the first, the stranger wore a badge displaying both the “Happy to Chat” message and a smiley image; in the second, the badge showed only a smiley image; and in the third, the stranger was not wearing a badge. The smile-only badge condition was included to distinguish between the influence of general positive affect cues and the explicit communicative signal conveyed by the “Happy to Chat” message itself. In addition, Age and gender were deliberately manipulated because prior research demonstrates that first impressions and willingness to engage socially are strongly shaped by demographic stereotypes associated with warmth and approachability (tel Stal et al., 2020). Including these variables enabled us to examine whether the Happy to Chat badge influences perception beyond established social expectations. Age was depicted at two levels: one showing a middle-aged individual, the other an elderly individual. Gender was also varied between two conditions: one depicting a male, the other a female. These manipulations resulted in a total of 12 distinct photo stimuli for each experimental condition (refer to Table 1 for details).

**Table 1:** Stimuli for each experimental condition based on badge presentation, age, and gender

	Badge presentation		
	Happy to Chat Badge	Simile only badge	No badge
Age & Gender			
Older male			
Mid-age male			
Older female			
Mid-age female			

### Preliminary Study

Before the main empirical test, we conducted a preliminary study to confirm that participants accurately perceived our manipulations of Badge Presentation, Age, and Gender. The online survey questionnaire was administered via YouGov (https://yougov.co.uk/), which randomly assigned participants to one of twelve conditions, ensuring each individual encountered only one stimulus (refer to Table 2 for details). This approach minimized selection bias and upheld methodological rigor and reliability in data collection (Campbell & Stanley, 2015; Shadish et al., 2002). A total of 528 participants (54.5% female; M_age_ = 49.46, *SD* = 18.10 range 18–94) participated.

**Table 2:** Descriptive statistics of survey participants

	*N* = 1738	
	N	%
*Gender:*		
Male	775	44.6
Female	963	55.4
*Region of residence:*		
North East	70	4.0
North West	229	13.2
Yorkshire and the Humber	182	10.5
East Midlands	164	9.4
West Midlands	179	10.3
East of England	210	12.1
London	219	12.6
South East	282	16.2
South West	203	11.7
*Area type:*		
Urban	1386	79.8
Town and Fringe	170	9.8
Rural	178	10.2
*Health/Disability status:*		
Yes, limited a lot	169	9.8
Yes, limited a little	346	20.1
No	1204	70.0
*Country of birth:*		
United Kingdom	1577	90.7
Other Countries	133	7.7
Prefer not to say	27	1.6
*Personal annual income:*		
Under £20,000	535	32
£20,000—£34,999	415	25
£35,000—£59,999	235	14
£60,000—£99,999	55	3
£100,000 and above	29	1.8
Prefer not to say	329	19.9

Through random allocation, each participant viewed a visual image and then confirmed their observations through four questions assessing the visibility and comprehension of the badge, as well as the perceived age and gender of the depicted person.

*Participants’ Perceptions of the Badge Presentation Manipulation*

In the “With Badge” condition, all participants correctly identified the presence of a badge whether it is the *Happy to Chat* badge or the smile only badge, while 96% in the “No Badge” condition observed its absence (*χ*^2^(1) = 489.32, *p* < .001). However, only 71% of participants in the *Happy to Chat* badge condition confirmed reading the “Happy to Chat” text on the badge. To address, this issue, in the follow up studies, we’ve increased the size of the badge by 15% and the pixels of the badges for better clarity.

*Participants’ Perceptions of the Age Manipulation*

In the "Older" conditions, 99% identified the older male or female in the photo as over 60, whereas in the "Mid-age" conditions, 93% perceived the male and female as aged 30–49 (*χ*^2^(6) = 517.14, *p* < .001).

*Participants’ Perceptions of the Gender Manipulation*

With the exception of one incorrect response, all participants correctly identified the gender as male or female in the respective "Male" or "Female" conditions, χ^2^(1) = 524.01, p < .001.

These results confirm that our manipulations were perceived as intended, validating the suitability of our stimuli for the main study.

### Experiment Design and Sample

The study employed a 3 (Badge Presentation: *Happy to Chat Badge* vs. *Simile Only Badge* vs. *No Badge*) X 2 (Age: *Older* vs. *Mid-age*) X 2 (Gender: *Male* vs. *Female*) between-subjects design. A between-subjects design was adopted because the study investigates spontaneous social perception in encounters with unfamiliar others. In everyday public settings, individuals typically encounter only one stranger at a time and form impressions without direct comparison to alternative conditions. Research on impression formation demonstrates that social judgments are often rapid and based on single exposures rather than comparative evaluation (Willis & Todorov, 2006). A within-subjects design, in which participants evaluate multiple badge variations, could introduce comparison effects and increase awareness of the experimental manipulation, thereby generating demand characteristics that influence responses (Orne, 2017). Presenting participants with a single stimulus therefore better approximates naturalistic first-impression formation and enhances ecological validity. Additionally, the between-subjects approach avoids carryover and testing effects whereby exposure to one condition may influence evaluation of subsequent targets (Shadish et al., 2002). For these reasons, a between-subjects design was considered most appropriate for examining how social signals function in realistic public encounters.

Similar to the preliminary study, the study employed an online survey questionnaire created and administered via the YouGov platform. Data collection took place over three months from October to December 2023, exclusively in England. A total of 1738 participants were randomly assigned and instructed to complete the online survey via YouGov (55.4% female; M_age_ = 49.08, *SD* = 18.15, range 18–90). Standard demographic information—including gender, age, region of residence, type of living area, health/disability status, country of birth, and personal income—was also collected as part of the default YouGov panel survey (see Table 2 for details).

### Procedures

Upon accessing the survey link, participants were randomly assigned to one of twelve conditions detailed in Table 1, each displaying a picture of a stranger of varying ages and genders. They were instructed to review the picture according to their assigned condition and answer questions assessing the person’s friendliness, interest in chatting, and trustworthiness. Participants were then asked if they would engage in various levels of interaction with this person, based on the following scenario:

“Imagine you are at a shopping centre that is bright and safe. You’ve just finished your shopping and have a bit of time to spare. If you see this person, would you…”

The different levels of interaction range from small social acknowledgments such as smiling, making eye contact, or nodding, to more active engagements like initiating a conversation, i.e. have a chat with this person. All responses were rated on a 5-point scale (see Table 3 for details).

**Table 3:** Measure items

Measures	All	
	Mean	*S.D*
**Ⅰ. Perceptions about a stranger**		
**Ⅰ-1. Friendliness**		
Do you think this person looks friendly? Very unfriendly-unfriendly-neither friendly or unfriendly-friendly-very friendly	3.83	0.64
**Ⅰ-2. Stranger’s Interest in Conversations**		
Do you think this person is interested in having a chat with others? Very uninterested-uninterested-neither uninterested or interested-interested-very interested	3.54	0.76
**Ⅰ-3. Trustworthiness**		
Do you think this person looks trustworthy? Very untrustworthy- untrustworthy- neither trustworthy or untrustworthy- trustworthy-very trustworthy	3.50	0.63
**Ⅱ. Behavioural intentions towards a stranger**		
Imagine you are at a shopping centre that is bright and safe. You’ve just finished your shopping and have a bit of time to spare. If you see this person, would you…		
**Ⅱ-1. Social Acknowledgment Intention**		
Smile, make eye contact and/or nod to this person? Very unlikely-unlikely-neither unlikely or likely-likely-very likely	3.60	1.06
**Ⅱ-2. Having a Chat Intention**		
Have a chat with this person? Very unlikely-unlikely-neither unlikely or likely-likely-very likely	2.83	1.15

*Notes:* N = 1738; *S.D.*: Standard Deviation. Responses were collected using a 5-point Likert scale, with descriptions provided below each statement

## Results

### Perceptions of a Stranger in a Stimuli Photo

*Friendliness*: Firstly, a 3 X 2 X 2 ANOVA was conducted to assess perceived friendliness toward the stranger in the photo, varying by badge presentation (*Happy to Chat Badge, Simile Only Badge*, *No Badge*), age, and gender. Significant main effects were found for badge presentation (*M*_*Happy to Chat badge*_ = 3.90, *SD* = .71 vs. *M*_*Simile Only Badge*_ = 3.86, *SD* = .58 vs. *M*_*No Badge*_ = 3.78, *SD* = .62; F(2, 1726) = 6.234, *p* = .002; *η*_*p*_^*2*^ = .007) supporting Hypothesis 1. Participants in the *Happy to Chat* badge condition showed the highest friendliness scores. Pairwise contrasts revealed this condition was significantly higher than No Badge condition (*p* < .001) but not significantly different from the Smile Only Badge condition (*p* = .213). Additionally, the Smile Only Badge condition was marginally higher than No Badge condition (*p* = .098). Significant main effects of age (F(1, 1726) = 13.356, p < .001; *η*_*p*_^*2*^ = .008) and gender (F(1, 1726) = 24.047, *p* < .001; *η*_*p*_^*2*^ = .014) revealed higher perceived friendliness toward older strangers (*M*_*Older*_ = 3.91, *SD* = .64) and female strangers (*M*_*Female*_ = 3.90, *SD* = .64) compared to mid-age strangers (*M*_*Mid-age*_ = 3.76, *SD* = .64) or male strangers (*M*_*Male*_ = 3.77, *SD* = .63) (See Table 4 for more details).

**Table 4:** ANOVA results: friendliness as a dependent variable

Source	Type III sum of squares	df	Mean square	F	Sig.	η_p_^2^
Corrected model	25.261^a^	11	2.296	5.775	0.000	.035
Intercept	22,240.440	1	22,240.440	55,929.182	0.000	.970
Badge presentation	4.958	2	2.479	6.234	0.002	.007
Age	5.311	1	5.311	13.356	0.000	.008
Gender	9.562	1	9.562	24.047	0.000	.014
Badge presentation * Age	1.958	2	0.979	2.461	0.086	.003
Badge presentation * Gender	2.271	2	1.135	2.855	0.058	.003
Age * Gender	0.005	1	0.005	0.012	0.913	.000
Badge presentation * Age * Gender	0.239	2	0.119	0.300	0.741	.000
Error	686.350	1726	0.398			
Total	26,248.000	1738				
Corrected total	711.611	1737				

a. R Squared = .031 (Adjusted R Squared = .029)

*Interest in conversation*

Interest in conversation was also examined using a 3 X 2 X 2 ANOVA (see Table 5). Significant effects for badge presentation (F(2, 1218) = 30.565, p < .001, ηp^2^ = .048) indicated that the stranger in the photo was perceived as having the highest interest in conversation when wearing the *Happy to Chat* badge (*M*_*Happy to Chat badge*_ = 3.84, *SD* = .86 vs. *M*_*Simile Only Badge*_ = 3.50, *SD* = .66 vs. *M*_*No Badge*_ = 3.44, *SD* = .74), supporting Hypothesis 2. Pairwise contrasts revealed that this condition was significantly higher than both the Smile Only badge (*p* < .001) and the No Badge condition (*p* < .001) Additionally, the Smile Only badge was not significantly different from the No Badge condition (*p* = .252). A significant effect of age was also found (F(1, 1218) = 22.733, p < .001; *η*_*p*_^*2*^ = .018) indicating that older strangers were perceived as showing greater interest in conversation (*M*_*Older*_ = 3.65, *SD* = .75), compared to mid-age strangers (*M*_*Mid-age*_ = 3.43, *SD* = .76).

**Table 5:** ANOVA results: interest in conversations as a dependent variable

Source	Type III sum of squares	df	Mean square	F	Sig.	η_p_^2^
Corrected model	51.200^a^	11	4.655	8.564	0.000	.072
Intercept	14,042.639	1	14,042.639	25,836.124	0.000	.955
Badge presentation	33.226	2	16.613	30.565	0.000	.048
Age	12.356	1	12.356	22.733	0.000	.018
Gender	1.093	1	1.093	2.010	0.156	.002
Badge presentation * Age	1.190	2	0.595	1.095	0.335	.002
Badge presentation * Gender	1.152	2	0.576	1.060	0.347	.002
Age * Gender	0.454	1	0.454	0.835	0.361	.001
Badge presentation * Age * Gender	0.064	2	0.032	0.059	0.943	.000
Error	662.016	1218	0.544			
Total	16,154.000	1230				
Corrected total	713.216	1229				

a. R Squared = .031 (Adjusted R Squared = .063)

*Trustworthiness*

Perceived trustworthiness was also analysed using a 3 X 2 X 2 ANOVA. The results revealed a significant badge effect (F(2, 1726) = 6.257, p = .002; *η*_*p*_^*2*^ = .007). The stranger wearing the *Happy to Chat* badge was perceived as most trustworthy (*M*_*Happy to Chat badge*_ = 3.57, *SD* = .67 vs. *M*_*Simile Only Badge*_ = 3.49, *SD* = .62 vs. *M*_*No Badge*_ = 3.45, *SD* = .60), supporting Hypothesis 3. Pairwise contrasts showed that the *Happy to Chat* condition was significantly higher than both the Simile Only Badge (*p* = .027) and the No Badge condition (*p* < .001). Also, the Simile Only Badge was not significantly different from No Badge condition statistically (*p* = .545). Nevertheless, significant effects of age (F(1, 1726) = 21.960, p < .001; *η*_*p*_^*2*^ = .013) and gender (F(1, 1726) = 3.849, p = .05; *η*_*p*_^*2*^ = .002) were found, indicating higher trustworthiness for older (*M*_*Older*_ = 3.57, *SD* = .62 vs. *M*_*Mid-age*_ = 3.43, *SD* = .63) and female strangers (*M*_*Female*_ = 3.52, *SD* = .65 vs. *M*_*Male*_ = 3.47, *SD* = .60) (See Table 6).

**Table 6:** ANOVA results: trustworthiness as a dependent variable

Source	Type III sum of squares	df	Mean square	F	Sig.	η_p_^2^
Corrected model	16.997^a^	11	1.545	3.984	0.000	.025
Intercept	18,432.643	1	18,432.643	47,521.437	0.000	.965
Badge presentation	4.854	2	2.427	6.257	0.002	.007
Age	8.518	1	8.518	21.960	0.000	.013
Gender	1.493	1	1.493	3.849	0.050	.002
Badge presentation * Age	0.086	2	0.043	0.111	0.895	.000
Badge presentation * Gender	0.999	2	0.500	1.288	0.276	.001
Age * Gender	0.985	1	0.985	2.541	0.111	.001
Badge presentation * Age * Gender	0.086	2	0.043	0.110	0.895	.000
Error	669.482	1726	0.388			
Total	21,935.000	1738				
Corrected total	686.479	1737				

a. R Squared = .031 (Adjusted R Squared = .019)

### Behavioural Intentions Towards a Stranger in a Stimuli Photo

*Social Acknowledgment Intentions*: we analysed whether participants are likely to smile, make eye contact and/or nod to the stranger in the photo using a 3 X 2 X 2 ANOVA. Significant main effects of badge presentation (F(2, 1726) = 6.498, p = .002; *η*_*p*_^*2*^ = .007) showed highest intentions for the *Happy to Chat* badge condition (*M*_*Happy to Chat badge*_ = 3.73, *SD* = 1.00 vs. *M*_*Simile Only Badge*_ = 3.60, *SD* = 1.00 vs. *M*_*No Badge*_ = 3.52, *SD* = 1.10). Pairwise contrasts indicated that this condition was significantly higher than both the Simile Only Badge (*p* = .041) and the No Badge (*p* < .001) conditions. Additionally, there is no significant difference from the Smile Only Badge condition to No Badge condition (*p* = .384). Significant main effects of age (F(1, 1726) = 14.671, p < .001; *η*_*p*_^*2*^ = .030) and gender (F(1, 1726) = 52.825, p < .001; *η*_*p*_^*2*^ = .008) were found, showing higher intentions to smile, make eye contacts or nods toward older (*M*_*Older*_ = 3.82, *SD* = .99 vs. *M*_*Mid-age*_ = 3.39, *SD* = 1.07) and female strangers (*M*_*Female*_ = 3.69, *SD* = 1.02 vs. *M*_*Male*_ = 3.52, *SD* = 1.08).

Interestingly, a significant interaction between badge presentation and age was found (F(2, 1726) = 3.561, *p* = .029). For older strangers depicted in the stimuli photos, participants showed higher intentions to smile, make eye contact, or nod, regardless of the badge presentation (F(2, 1726) = 2.286, p = .102; *η*_*p*_^*2*^ = .003; *M*_*Happy to Chat badge*_ = 3.91, *SD* = .95 vs. *M*_*Smile Only Badge*_ = 3.71, *SD* = .96 vs. *M*_*No Badge*_ = 3.81, *SD* = 1.03). Conversely, for middle-aged strangers depicted in the stimuli photos, the lowest intentions to engage were observed in the No Badge condition. relative to the other two badge presentation conditions (F(2, 1726) = 7.995, p < .001; *η*_*p*_^*2*^ = .009; *M*_*Happy to Chat badge*_ = 3.55, *SD* = 1.02 vs. *M*_*Simile Only Badge*_ = 3.48, *SD* = 1.03 vs. *M*_*No Badge*_ = 3.25, *SD* = 1.10). Altogether, hypothesis 4 was supported (See Table 7).

**Table 7:** ANOVA results: social acknowledgment intention as a dependent variable

Source	Type III sum of squares	df	Mean square	F	Sig.	η_p_^2^
Corrected model	124.130^a^	11	11.285	10.755	0.000	.064
Intercept	19,676.931	1	19,676.931	18,753.899	0.000	.916
Badge presentation	13.636	2	6.818	6.498	0.002	.007
Age	55.425	1	55.425	52.825	0.000	.030
Gender	15.393	1	15.393	14.671	0.000	.008
Badge presentation * Age	7.472	2	3.736	3.561	0.029	.004
Badge presentation * Gender	5.105	2	2.552	2.433	0.088	.003
Age * Gender	0.167	1	0.167	0.159	0.690	.000
Badge presentation * Age * Gender	2.008	2	1.004	0.957	0.384	.001
Error	1810.950	1726	1.049			
Total	24,461.000	1738				
Corrected total	1935.080	1737				

a. R Squared = .031 (Adjusted R Squared = .058)

*Intention to Have a Chat*: A 3 × 2 × 2 ANOVA was conducted to examine participants’ intention to engage in conversation. The results revealed no significant effect of badge presentation, indicating that participants’ intention to initiate a conversation did not significantly differ based on whether the stranger in the photo was wearing the *Happy to Chat* badge, the smile-only badge, or no badge, thus rejecting hypothesis 5 (See Table 8).

**Table 8:** ANOVA results: intention to have a chat as a dependent variable

Source	Type III sum of squares	df	Mean square	F	Sig.	η_p_^2^
Corrected model	70.364^a^	11	6.397	4.956	0.000	.031
Intercept	12,055.704	1	12,055.704	9339.750	0.000	.844
Badge presentation	0.808	2	0.404	0.313	0.731	.000
Age	36.942	1	36.942	28.620	0.000	.016
Gender	1.210	1	1.210	0.938	0.333	.001
Badge presentation * Age	8.320	2	4.160	3.223	0.040	.004
Badge presentation * Gender	1.091	2	0.546	0.423	0.655	.000
Age * Gender	0.229	1	0.229	0.177	0.674	.000
Badge pPresentation * Age * Gender	0.821	2	0.410	0.318	0.728	.000
Error	2227.913	1726	1.291			
Total	16,260.000	1738				
Corrected total	2298.276	1737				

a. R Squared = .031 (Adjusted R Squared = .024)

Additionally, intention to have a chat was not significantly influenced by gender, suggesting that participants’ willingness to initiate a conversation did not differ based on whether the stranger was male or female. However, the results showed that intention to have a chat was significantly influenced by age (F(1, 1726) = 28.620, p < .001; *η*_*p*_^*2*^ = .016), signifying that participants’ higher willingness to have a chat with older strangers (*M*_*Older*_ = 3.02, *SD* = 1.15) than middle-age strangers (*M*_*Mid-age*_ = 2.65, *SD* = 1.13). Interestingly, a significant badge by age interaction effect was observed (F(2, 1726) = 3.223, p = .04; *η*_*p*_^*2*^ = .004). However, the results indicated that the nudge effect of the badge on willingness to have a chat was weaker within each age group compared to its effect on willingness to engage in social acknowledgements (e.g., smiling, making eye contact, or nodding). Specifically, among older strangers, no significant differences were found across three badge conditions (F(2, 1726) = 2.203, p = .111; *η*_*p*_^*2*^ = .003; *M*_*Happy to Chat badge*_ = 2.99, *SD* = 1.18 vs. *M*_*Simile Only Badge*_ = 2.89, *SD* = 1.08 vs. *M*_*No Badge*_ = 3.09, *SD* = 1.15). Likewise, no significant differences were found among badge conditions for middle-aged strangers (F(2, 1726) = 1.388, p = .250; *η*_*p*_^*2*^ = .002; *M*_*Happy to Chat badge*_ = 2.72, *SD* = 1.12 vs. *M*_*Smile Only Badge*_ = 2.71, *SD* = 1.09 vs. *M*_*No Badge*_ = 2.59, *SD* = 1.14).

The absence of badge effects in nudging participants to initiate a conversation may reflect the differing levels of engagement required when interacting with strangers—ranging from simple social acknowledgements to having an actual conversation. Behaviours such as smiling, making eye contact, or nodding function as polite social signals that acknowledge another’s presence within a community. In contrast, initiating a chat requires a more proactive and deliberate effort, involving greater cognitive and emotional engagement. As such, the willingness to engage in conversation may be more strongly influenced by the characteristics of the potential conversation partner—such as their age—than by the presence of a badge.

These findings suggest that the influence of badges as social nudges is more effective at lower levels of engagement (e.g., minimal acknowledgements) and tends to diminish when higher levels of interaction are required. When interactions demand greater effort, individuals may rely more on pre-existing social associations, which can override the subtle prompting of the badge.

To further understand these dynamics, we conducted additional contrast analyses using age as an independent variable and badge presentation as a moderator. Specifically, the results show that within the *Happy to Chat* Badge condition, participants exhibited significantly higher intentions to chat with older strangers than middle-aged strangers (F(2, 1726) = 7.313, p = .007; *η*_*p*_^*2*^ = .004; *M*_*Older*_ = 3.00, *SD* = 1.18 vs. *M*_*Mid-age*_ = 2.72, *SD* = 1.12). Similarly, in the No Badge condition, intentions were significantly higher toward older strangers than middle-aged strangers (F(2, 1726) = 41.909, p < .001; *η*_*p*_^*2*^ = .024; *M*_*Older*_ = 3.09, *SD* = 1.15 vs. *M*_*Mid-age*_ = 2.60, *SD* = 1.14). However, no significant age differences were observed in intentions to have a chat, in the Smile Only Badge condition (F(2, 1726) = 1.984, p = .159; *η*_*p*_^*2*^ = .001; *M*_*Older*_ = 2.89, *SD* = 1.08 vs. *M*_*Mid-age*_ = 2.71, *SD* = 1.09).

### Post-Hoc Power Analysis

To address potential concerns that the large sample size used in our study (N = 1,738) may inflate statistical significance and lead to potentially misleading interpretations (Maier & Lakens, 2022), we conducted a post hoc power analysis using G*Power, a widely recognised tool for assessing statistical power and determining appropriate sample sizes in experimental research (Prajapati et al., 2010). This analysis aimed to ascertain the statistical power of our tests, assuming that the effect sizes estimated from our collected data are accurate (Lakens, 2022; Lenth, 2007; Zumbo & Hubley, 1998). We set parameters for an effect size f = 0.01, which corresponds to a small effect size, based on computed effect sizes (ηp^2^) ranging from 0.002 to 0.48, indicative of small to medium effects in our dataset (i.e., Serdar et al., 2021). Following recommendations by Maier and Lakens (2022), we adjusted the alpha level to α = 0.01 to minimize the risk associated with the large sample size. We also accounted for a total of 1738 participants, numerator degrees of freedom (df) of 2, and 12 groups. The analysis yielded a power of 0.89, which surpasses the conventional threshold of 0.8 and falls within the conventionally acceptable and desirable range (Serdar et al., 2021). This confirms that the power is adequate to address concerns regarding potential overestimation of effects due to the large sample size.

## Discussions & Implications

This study examined whether a simple social signal—the *Happy to Chat* badge—can function as a universal social nudge that reshapes how strangers are perceived in everyday public encounters. Rather than positioning the badge as an intervention aimed at individuals experiencing loneliness, our findings suggest that its primary role lies in making openness to interaction socially visible and temporarily legitimate. The results indicate that when willingness to engage is signalled explicitly, strangers are perceived as friendlier, more trustworthy, and more interested in conversation, thereby lowering initial psychological barriers to social acknowledgement. The following discussion interprets these findings in relation to the proposed mechanisms of social nudging and everyday interaction.

Firstly, wearing a Happy to Chat badge increases the likelihood of being perceived as a ‘good stranger’ (Zeeb & Joffe, 2021)—someone who is friendly, approachable, and potentially enriching to engage with. As ‘good strangers’ are typically associated with positive and meaningful social encounters, badge wearers may be more warmly received during interactions with others. Secondly, Schroeder et al. (2022) note that people often avoid initiating conversations with strangers due to the mistaken belief that others are disinterested in engaging. Our findings provide empirical evidence that such misperceptions can be partially reduced when willingness to engage is made socially visible: individuals wearing a *Happy to Chat* badge are perceived as significantly more interested in having a conversation than those without one. This suggests the badge functions as a clear social signal of openness, helping reduce one of the key psychological barriers to initiating conversations (Schroeder et al., 2022). When intentions to connect are made visible, it becomes easier for individuals to engage with one another (Atir et al., 2023).

Our findings also confirm that people wearing the *Happy to Chat* badges are more likely to receive smiles, eye contacts, or nods than those without badges or those only wearing a smile badge (Hypothesis 4). While the results show that, compared to middle-aged individuals, older people are generally more likely to receive social acknowledgements, wearing a *Happy to Chat* badge can further increase their likelihood of receiving such acknowledgements—such as smiles, eye contact, or nods from others—when out and about. This supports the effectiveness of the *Happy to Chat* badge as a social nudge that encourages social acknowledgement and may contribute to enhanced feelings of connection within communities—as it successfully encourages others to acknowledge one’s social presence through nonverbal cues such as smiles, eye contact, and head nods. Even brief everyday acknowledgements can foster a sense of connection and belonging (Algoe, 2012; Yen et al., 2024). This finding offers encouraging news for individuals experiencing social isolation—wearing a *Happy to Chat* badge can increase the likelihood of receiving friendly non-verbal cues from others in the community, such as smiles, eye contact, and head nods. Being socially acknowledged in this way can help reduce feelings of loneliness and social isolation (Power et al., 2019) and contribute positively to subjective well-being (Gunaydin et al., 2020; Sandstrom & Dunn, 2014).

Finally, our findings show no significant difference in participants’ intention to approach the person in the photo for a chat across the three badge conditions. This highlights a limitation of the nudge—simply wearing a *Happy to Chat* badge does not guarantee that one will be approached for conversation, even if the badge enhances perceptions of friendliness, trustworthiness, and openness. Importantly, the findings suggest that social nudges may influence different aspects of social interaction unevenly, shaping perceptions and low-effort acknowledgements more readily than higher-effort behaviours such as initiating conversation. While the badge significantly increased favourable perceptions and low-effort acknowledgements such as smiling or eye contact, it did not independently increase willingness to initiate conversations. This pattern is consistent with broader cultural norms in England, where spontaneous conversations with strangers are not always common and social boundaries around initiating contact tend to be more reserved (Grierson, 2016). Within such a context, norm-based interventions may first influence perception and social permission before translating into higher-effort behaviours. From this perspective, the *Happy to Chat* badge functions less as a direct conversation trigger and more as an enabling infrastructure that makes everyday friendliness socially acceptable (Yen & Özbilgin, 2025). As a nudge rather than a promotional intervention, the badge requires minimal investment and is therefore expected to generate incremental rather than large immediate behavioural effects.

Several additional factors may help explain the absence of badge effects on willingness to initiate conversations. Initiating a conversation requires greater cognitive and emotional effort than offering minimal acknowledgements such as smiling or nodding, meaning that behavioural escalation may depend more strongly on individual readiness than on external cues. Time availability may also play a role, as individuals balancing work and family responsibilities may be less inclined to engage spontaneously compared to those with greater discretionary time (Yen et al., 2024). Furthermore, consistent with prior research (Glück & Bluck, 2013; Luong et al., 2011), older strangers may be perceived as more approachable or socially rewarding conversational partners, helping explain the stronger age effects observed independent of badge presentation.

Taken together, these findings contribute to nudge theory by demonstrating that social nudges may function less as direct behaviour-change tools and more as mechanisms that reshape perceived social norms and interactional permission. By signalling openness rather than prescribing behaviour, universal social signals such as the *Happy to Chat* badge may help normalise everyday sociability and lower psychological barriers to connection in public spaces.

## Limitations and Future Research Suggestions

While this study provides valuable insights, several limitations warrant discussion.

Firstly, the visual stimuli were necessarily simplified representations of strangers. All depicted individuals were white and appeared relatively attractive – despite our prompts for average physical appearance in generating the AI images, which does not fully reflect the demographic and appearance diversity of everyday public encounters in England. Prior research shows that attractiveness and demographic cues influence social perception and may therefore affect responses independently of the badge manipulation. Moreover, static images cannot fully capture the dynamic nature of real-world interactions. Future research should therefore employ more diverse stimuli and field-based studies in natural public environments to enhance ecological validity (Shin, 2025; Yen et al., 2024).

Secondly, the study examined only one version of the Happy to Chat badge. While this ensured experimental control, future research may explore whether different visual presentations influence the strength of the social signal. Thirdly, several constructs were measured using single-item indicators to minimise participant burden across multiple experimental conditions. Although appropriate for exploratory research, future studies could employ validated multi-item scales to enhance measurement reliability and construct precision. Fourthly, participants evaluated hypothetical scenarios rather than engaging in real interactions. Behavioural intentions do not always translate into actual behaviour in natural settings where contextual and interpersonal dynamics unfold in real time. Field experiments examining observed behaviour would therefore provide an important next step.

Finally, cultural norms shape expectations surrounding interaction with strangers. As this study was conducted in England, future research should examine whether similar social nudges operate differently across cultural contexts characterised by varying norms of interpersonal openness. For example, in Latin America, happiness is closely linked to the abundance of close, warm, and genuine interpersonal relationships across various domains of life (Rojas, 2024). Examining the badge’s impact in collectivist cultures—where social norms around engagement and interpersonal openness differ—could offer valuable insights into the generalisability and cultural sensitivity of such interventions. While prior research shows that interactions with strangers can enhance well-being (Diener & Seligman, 2002; Mehl et al., 2010), future studies could further investigate which specific dimensions of well-being (e.g., cognitive, affective, social) are most influenced by these interactions, how cultural context moderates this impact, and whether such benefits are sustained over time.

Taken together, these limitations highlight important directions for advancing research on universal social signals in public life. Replication across diverse cultural contexts and behavioural field settings will further clarify how social nudges contribute to the normalisation of everyday social connection.

## Data Availability

Upon publication, the data will be made available on the project website (https://www.trusttracker.org/data).
